# Birth rates among male cancer survivors and mortality rates among their offspring: a population-based study from Sweden

**DOI:** 10.1186/s12885-016-2236-y

**Published:** 2016-03-08

**Authors:** Siau-Wei Tang, Jenny Liu, Lester Juay, Kamila Czene, Hui Miao, Agus Salim, Helena M Verkooijen, Mikael Hartman

**Affiliations:** Department of Surgery, Yong Loo Lin School of Medicine, 1E Kent Ridge Road, National University Health System, Singapore, 119228 Singapore; Saw Swee Hock School of Public Health, National University of Singapore, 16 Medical Drive, Singapore, 117597 Singapore; Department of Medical Epidemiology and Biostatistics, Karolinska Institute, SE-171 77 Stockholm, Sweden; Department of Mathematics and Statistics, La Trobe University, Bundoora, VIC 3086 Australia; Department of Radiology, University Medical Center, Heidelberglaan 100, 3584 CX Utrecht, The Netherlands

**Keywords:** Fathers, Cancer survivors, Birth rates, Mortality, Offspring

## Abstract

**Background:**

With improvements in treatment of cancer, more men of fertile age are survivors of cancer. This study evaluates trends in birth rates among male cancer survivors and mortality rates of their offspring.

**Methods:**

From the Swedish Multi-generation Register and Cancer Register, we identified 84,752 men ≤70 years with a history of cancer, for which we calculated relative birth rates as compared to the background population(Standardized Birth Ratios, SBRs). We also identified 126,696 offspring of men who had cancer, and compared their risks of death to the background population(Standardized Mortality Ratio, SMRs). Independent factors associated with reduced birth rates and mortality rates were estimated with Poisson modelling.

**Results:**

Men with a history of cancer were 23 % less likely to father a child compared to the background population(SBR 0.77, 95 % Confidence Interval[CI] 0.75–0.79). Nulliparous men were significantly more likely to father a child after diagnosis (SBR 0.81, 95 % CI 0.79–0.83) compared to parous men (SBR 0.68, 95 % CI 0.66–0.74). Cancer site(prostate), onset of cancer during childhood or adolescence, parity status at diagnosis(parous), current age(>40 years) and a recent diagnosis were significant and independent predictors of a reduced probability of fathering a child after diagnosis.

Of the 126,696 children born to men who have had a diagnosis of cancer, 2604(2.06 %) died during follow up. The overall mortality rate was similar to the background population(SMR of 1.00, 95 %CI 0.96–1.04) and was not affected by the timing of their birth in relation to father’s cancer diagnosis.

**Conclusion:**

Male cancer survivors are less likely to father a child compared to the background population. This is influenced by cancer site, age of onset and parity status at diagnosis. However, their offspring are not at an increased risk of death.

## Background

With early diagnosis, advancing treatment and improved survival rates of childhood and early adult cancer, an increasing proportion of boys and young men with cancer are more likely to survive their disease to reach reproductive ages [1]. This has led to an increased interest in the quality of life and reproductive potential of this group of cancer survivors. Some forms of cancer may directly affect fertility through adverse effects on the physiology of the male reproductive organs or endocrine glands; eg. Testicular cancer. Loco-regional or systemic treatment procedures such as pelvic surgery, radiotherapy or chemotherapy may induce temporary or permanent infertility in men through the disruption of ejaculatory functions or cytotoxic effects on spermatogenesis [2, 3].

The advances in medicine have had a contrasting effect on fertility in cancer survivors. On one hand, newer chemotherapeutic treatment regimes (eg. the use of procarbazine with alkylating agents in Hodgkin’s disease) or gonadal/whole body irradiation has a higher chance of sterilising patients [4, 5]. However, the development of fertility preservation techniques in men, such as semen cryopreservation and testicular sperm extraction; in combination with artificial reproductive techniques of in-vitro fertilisation (IVF) or intra-cystoplasmic sperm injection (ICSI) have offered men a possibility of parenthood after cancer [4, 5].

Psychologically, the diagnosis of cancer may increase the value placed on family and the importance of parenthood for the cancer survivors [6, 7]. However, this may be contradicted with his own uncertainties of his cancer prognosis, a perceived risk of passing the cancer susceptibility on to his offspring, treatment-related harm to the offspring and other social and cultural influences. Studies have shown that cancer patients are less likely to father a pregnancy after their disease as compared to their siblings [8, 9].

Various studies have not shown any adverse pregnancy outcomes for the partners of male childhood cancer survivors [10, 11]. However, few studies have been conducted in adult male cancer survivors. It is unclear if the exposure to (the effects of) diagnostic investigations, radiation therapy, and systemic treatment before and around the time of birth may have any adverse effects on spermatogenesis and the subsequent wellbeing of the offspring.

In this study, we aim to evaluate trends in birth rates among Swedish male cancer survivors by age and over time, as well as the factors which independently affect their probability of fatherhood after diagnosis. We also aim to assess the mortality risks in offspring of these men with a history of cancer in relation to timing of birth and cancer site.

## Methods

### Study design

We used the Multi-Generation Register(MGR) [12], the Swedish Cancer Register [13], the Cause of Death Register, and the Migration Register for this study. The unique national registration numbers accorded for each Swedish individual was used as a linkage between the registries and the Censuses of 1960, 1970, 1980, and 1990, to obtain further information on the socioeconomic status of each Swedish citizen. Information was extracted on cancer site as defined by the International Classification of Diseases, Revision 7 code (ICD7) [14]. Socioeconomic status was estimated based on information about the highest level of employment in the household given in the Censuses and was categorized into five groups; blue collar workers, white collar workers, self-employed workers, farmers and unclassified. In total, this database contains more than 11 million individuals, belonging to around three million families and included more than a million individuals with cancer diagnosed between 1958 and 2001 [12]. The study design was approved by the Regional Ethics Review Board at Karolinska Institutet, Sweden whereby the need for individual consent from the participants was deemed unnecessary.

### Birth rates among male cancer survivors

Our study base consisted of all men aged 16 to 70 years, born after 1931, who were alive between 1961 and 2002 and present in the Multi-Generation Register. The partners or spouse of these men were identified and tracked for up to 10 months after the men have passed away/emigrated to ensure all information of their offspring were included. For each man, we ascertained the number and dates of live child births (multiple pregnancies were counted as one event/birth), with their partners or spouse, socioeconomic status, date of cancer diagnosis (if present) and followed them until death, emigration or end of follow up (31st December 2002), whichever came first. End of follow-up was chosen at 31st December 2002, where records of cancer, death, migration and socioeconomic status were all available on the numerous registers. For men diagnosed with cancer, we calculated the proportion and relative probability of fathering an offspring after diagnosis.

**Fig. 1:**
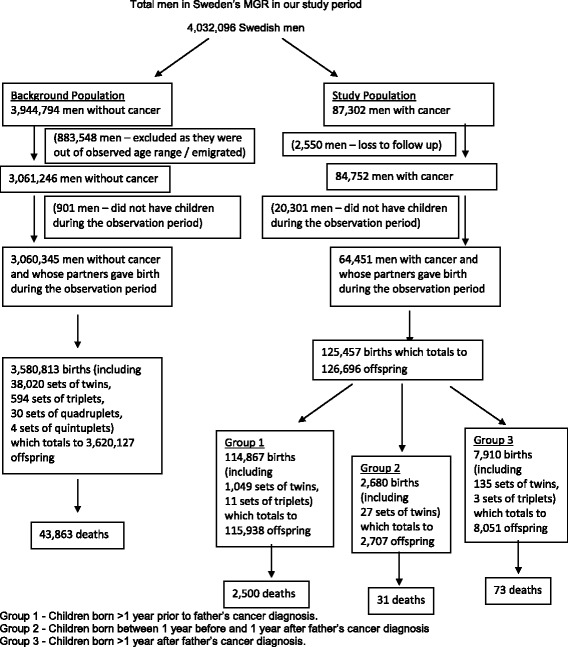
Flowchart of the Swedish male population in the Multi-Generation Register (MGR) aged 16 to 70 years, born after 1931, who were alive in 1961

We included all births >1 year after diagnosis, as most patients would have received complete information on treatment plan and prognosis by then, with the decision to have an offspring considered with the knowledge of the cancer diagnosis. For the background population, cancer-free men in the background population were matched to the male cancer survivors according to attained age and year of birth, and the number of live child births of the spouse of these men in the background population was then ascertained. Using the above criteria, there were 4,032,096 Swedish men in the Swedish MGR, where 87,302 men had a history of cancer (Fig. 1). Over the study period, 2550 participants (0.03 %) were lost to follow-up and 883,548 (22.4 %) were excluded from the study population as they were out of the observed age range of 16 to 70 years old or had emigrated. Missing data is equally likely in both the male cancer survivors and the background population which is why we chose to use standardized birth ratio to handle the non-differential bias.

### Mortality rate among offspring of male cancer survivors

For the offspring of fathers with a history of cancer we extracted information on date of birth, date of death and cause of death. Each individual offspring in multiple pregnancies was counted separately. 126, 696 offspring of 64,451 men in the study population with cancer and whose partners gave birth during the observation period were identified and followed up for a median of 32.3 years (range 0–42.9 years). Causes of death were regrouped as congenital anomalies (ICD7: 750–759), perinatal conditions (ICD7: 760–776),neoplasms (ICD7: 140–239) and others (i.e. all remaining causes combined). Offspring were followed from birth until death, emigration, or end of follow-up (31^st^ December 2003), whichever came first.

In order to investigate the association between timing of birth in relation to the father’s cancer diagnosis, we categorized offspring into three groups: offspring born more than 1 year before their father’s cancer diagnosis (Group 1), offspring born between 1 year before and 1 year after their father’s diagnosis (Group 2), and offspring born more than 1 year after their father’s cancer diagnosis (Group 3). The rationale behind these (arbitrary) cut off levels was that children born more than 1 year before their father’s diagnosis were not exposed to the cancer process itself, nor to the diagnostic investigations, or treatment. Children born around their father’s diagnosis may have been conceived with sperm that were directly exposed to the cancer process itself, the diagnostic investigations (involving ionizing radiation), and the cancer treatment. Finally, children born more than 1 year after their father’s diagnosis have not been directly exposed to cancer treatment, but mutagenic effects of cancer treatment or damage to the reproductive organs in the father may still affect their outcome.

### Statistical analysis

Relative birth rates were expressed as a Standardized Birth Ratios (SBRs), i.e. the ratio of observed births in our study group to the expected number of births, based on birth rates in the background population. Person-years at risk of giving birth were calculated from 1 year after the time of cancer diagnosis or 16^th^ birthday, whichever came later; until 10 months after date of death or emigration, 17^th^ birthday or end of follow up (31st December 2003), whichever came first. Background birth rates were derived using birth rates for all men present in the Multi-Generation Register [12]. The expected number of births was calculated by multiplying the 5-year age- and calendar period specific birth rates in the background population by correspondingly stratified person-years at risk and summing all the products. We then compared relative birth rates by age at onset (childhood-below the age of 13 years-, adolescence−13 to 18 years-and adulthood > 18 years), attained age, year and parity status at diagnosis, cancer site, time since diagnosis, attained year, and socioeconomic status.

Relative risks of death in offspring were expressed as Standardized Mortality Ratios(SMRs), i.e. the ratio of observed to expected number of deaths. Person-years were calculated as the time from the child’s birth to death, emigration, or end of follow up (31^st^December 2003), whichever came first. Background mortality rates were derived using mortality rates of 3,620,127 offspring of 3,060,345 men present in the Multi-Generation Register (Fig. 1). The expected number of deaths was calculated by multiplying the 5-year age and calendar period specific mortality rates in the background population by correspondingly stratified person-years at risk in the cohort and summing all the products. We calculated overall SMRs by timing of birth in relation to father’s diagnosis and stratified attained age of the offspring and cancer site.

A multivariate Poisson regression modelling was performed on data from the study population only, to identify independent predictors of fatherhood after diagnosis and the mortality of the offspring of cancer survivors. The relative risks of fatherhood were expressed as Birth Rate Ratios (BRRs), which were adjusted for parity status at diagnosis, cancer site, age and year of diagnosis, time since diagnosis, calendar period, attained age and socioeconomic status. The BRRs was also used to estimate whether factors linked with mortality in the offspring on univariate analysis remained significantly associated after adjustment for father’s age at childbirth, mother’s age at childbirth, attained age of child, calendar year and socioeconomic status. Relative risks were expressed as Mortality Rate Ratios (MRRs). Finally, we compared causes of death in offspring of fathers with cancer to causes of death of children in the background population. Chi-square and Kruskal-Wallis tests were used to test for differences in the distribution of causes of death. Data preparation and analysis was done with SAS^®^ version 9.2(SAS Institute Inc., Cary, North Carolina).

## Results

The study population included 3,145,998 Swedish men, of whom 84,752 (2.8 %) were diagnosed with cancer before age 70 years (Fig. 1). Most of the men (95.7 %) were diagnosed with cancer in adulthood (19–70 years), with the commonest site being digestive tract (n = 14,817) and prostate (n = 11,281) (Table 1). Among the cancer survivors (group 3), 4973 (5.9 %) men had partners who gave birth to a total of 7910 births 1 year after their diagnosis. A higher proportion of men who were diagnosed in childhood (19.5 %) and adolescence (22.8 %) fathered a child after the diagnosis, compared with men diagnosed in adulthood (5.2 %).

**Table 1:** Men diagnosed with cancer in Sweden 1960–2002 with information on subsequent birth(s) by age and year of diagnosis, parity status at diagnosis and cancer site

	ICD7 codes	Number of men with previous cancer	Number of men* with births after cancer diagnosis	Total number of births after cancer diagnosis	Proportion of men whose partners gave birth >1 year after cancer diagnosis
Overall	–	84752	4973	7910	5.9 %
*Age at diagnosis*					
0–12 (Childhood)	–	1700	332	610	19.5 %
13–18 (Adolescence)	–	1970	449	891	22.8 %
19–70 (Adult)	–	81082	4192	6409	5.2 %
*Year of diagnosis*					
1958 to 1968	–	1854	769	1366	41.5 %
1969 to 1979	–	6289	1265	2271	20.1 %
1980 to 1990	–	18332	1739	2751	9.5 %
1991 to 2001	–	58277	1200	1522	2.1 %
*Parity Status at diagnosis*					
Nulliparous	–	22296	3258	5706	14.6 %
Parous	–	62456	1715	2204	2.7 %
*Cancer site*					
Digestive	150.0–158.9	14817	350	544	2.4 %
Prostate	177.0–177.9	11281	4	4	0.0 %
Others^a^	180.0–181.9; 191.0–191.9; 209.0–209.9	10285	377	611	3.7 %
Bone	196.0–200.9	8764	588	993	6.7 %
Brain & eye	192.0–193.9	7212	715	1188	9.9 %
Hematopoietic	201.0–208.9	7049	575	935	8.2 %
Thoracic	162.0–164.9	6529	55	91	0.8 %
Melanoma skin	190.0–190.9	6489	679	1032	10.5 %
Reproductive organs	178.0–179.9	5276	1052	1555	19.9 %
Head & neck	140.0–148.9; 160.0–161.9	3706	167	280	4.5 %
Thyroid	194.0–195.9	3224	408	674	12.7 %
Breast	170.0–170.9	120	3	3	2.5 %
*Socio-economic Status*					
Blue Collar Workers	–	32294	1884	2923	5.8 %
White Collar Workers	–	33837	2072	3436	6.1 %
Self-employed	–	5723	211	338	3.7 %
Farmers	–	1666	81	146	4.9 %
Unclassified	–	11232	725	1067	6.5 %

*Abbreviations*: *ICD7* International Classification of Diseases, Revision 7 Codes

*Father’s age at birth of child is 16–70 years

^a^ Including malignant neoplasm of Kidney, Bladder/urinary organs, and other (non-melanoma) malignant neoplasm of the skin

Men diagnosed with cancer were 23 % less likely to have a child (SBR 0.77, 95 % CI 0.75–0.79) compared to the background population (Table 2). This remained fairly consistent over calendar time. Men who were diagnosed in childhood had a significantly lower birth rate (SBR 0.62, 95 % CI 0.57–0.67) than men diagnosed in adulthood (SBR 0.80, 95 % CI 0.78–0.82) when compared to the background population. Survivors of skin, thoracic cancer and head and neck had a birth rate similar to the background population (SBR 0.98 [0.92–1.04],0.92 [0.74–1.11] and 0.90 [95 % CI 0.80–1.01] respectively); whilst survivors of prostate, brain and eye, and hematopoietic cancers had a significant decrease in their birth rates (SBR 0.24 [95 % CI 0.06–0.54], 0.65 [0.62–0.69] and 0.66 [0.62–0.70] respectively) compared to the background population.

**Table 2:** Standardised Birth Ratios (SBRs) of men diagnosed with cancer in Sweden 1960–2002 with information on subsequent birth(s) >1 year after cancer diagnosis

	Observed	Expected	SBR
Overall	7910	10252	**0.77 (0.75–0.79)**
*Attained Age*			
16 to 25	682	999	**0.68 (0.63–0.73)**
26 to 30	2160	2768	**0.78 (0.75–0.81)**
31 to 35	2448	3149	**0.78 (0.75–0.81)**
36 to 40	1572	1929	**0.81 (0.78–0.86)**
41 to 45	678	866	**0.78 (0.72–0.84)**
46 to 70	370	541	**0.68 (0.62–0.76)**
*Age at diagnosis*			
Childhood (0 to 12 years)	610	986	**0.62 (0.57–0.67)**
Adolescence (13 to 18 years)	891	1235	**0.72 (0.67–0.77)**
Adulthood (19 to 70 years)	6409	8031	**0.80 (0.78–0.82)**
*Year of diagnosis*			
1958 to 1968	1366	1708	**0.80 (0.76–0.84)**
1969 to 1979	2271	2950	**0.77 (0.74–0.80)**
1980 to 1990	2751	3621	**0.76 (0.73–0.79)**
1991 to 2001	1522	1973	**0.77 (0.73–0.81)**
*Cancer site*			
Head & neck	280	312	0.90 (0.80–1.01)
Digestive	544	611	**0.89 (0.82–0.97)**
Thoracic	91	99	0.92 (0.74–1.11)
Breast	3	6	0.54 (0.10–1.32)
Prostate	4	17	**0.24 (0.06–0.54)**
Reproductive organs	1555	2201	**0.71 (0.67–0.74)**
Melanoma skin	1032	1052	0.98 (0.92–1.04)
Brain & eye	1188	1820	**0.65 (0.62–0.69)**
Thyroid	674	793	**0.85 (0.79–0.92)**
Bone	993	1211	**0.82 (0.77–0.87)**
Hematopoietic	935	1421	**0.66 (0.62–0.70)**
Others	611	711	**0.86 (0.79–0.93)**
*Parity Status at diagnosis*			
Nulliparous	5706	7032	**0.81 (0.79–0.83)**
Parous	2204	3221	**0.68 (0.66–0.71)**
*Time since diagnosis*			
1 to 5 years	3738	4734	**0.79 (0.76–0.82)**
6 to 10 years	2090	2589	**0.81 (0.77–0.84)**
11 to 20 years	1558	2156	**0.72 (0.69–0.76)**
21 to 45 years	524	773	**0.68 (0.62–0.74)**
*Socio-economic Status*			
Blue Collar Workers	2923	4183	**0.70 (0.67–0.72)**
White Collar Workers	3436	3869	**0.89 (0.86–0.92)**
Self-employed	338	399	**0.85 (0.76–0.94)**
Farmers	146	142	1.03 (0.87–1.20)
Unclassified	1067	1658	**0.64 (0.61–0.68)**

Bold number units indicate a significant decrease in SBRs

Men who were parous at the point of cancer diagnosis had a lower birth rate compared to those who were nulliparous (SBR 0.68 [0.66–0.74], 0.81 [95 % CI 0.79–0.83] respectively). This was particularly pronounced in parous men who were diagnosed with prostate, thoracic and haematopoietic cancers (SBR 0.22 [0.04–0.53], 0.48 [0.40–0.88] and 0.53 [0.46–0.61] respectively) (Table 3). In contrast, men who were nulliparous at point of diagnosis of thoracic, skin, head and neck and digestive cancers had birth rates which were similar to the background population (SBR 1.20 [0.94–1.49], 1.10 [1.01–1.18], 1.06 [0.92–1.22], 0.97 [0.87–1.08] respectively). However, nulliparous men who were diagnosed with brain and eye, haematopoietic and reproductive organs cancer still had significantly lower birth rates compared to the background population (SBR 0.65 [0.60–0.69], 0.70 [0.65–0.75], 0.75 [0.68–0.77] respectively).

**Table 3:** Standardized Birth Ratios (SBRs) of men whose partners gave birth more >1 year after cancer diagnosis by parity status at cancer diagnosis and cancer site

Cancer site	Men whose partners gave birth >1 year after cancer diagnosis	
	*Nulliparous*	*Parous*
Head & neck	1.06 (0.92–1.22)	**0.67 (0.54–0.82)**
Digestive	0.97 (0.87–1.08)	**0.77 (0.67–0.89)**
Thoracic	1.20 (0.94–1.49)	**0.48 (0.29–0.72)**
Breast	0.56 (0.00–2.19)	0.53 (0.05–1.51)
Prostate	0.39 (0.00–1.53)	**0.22 (0.04–0.53)**
Reproductive organs	**0.75 (0.71–0.80)**	**0.61 (0.56–0.67)**
Melanoma skin	1.10 (1.01–1.18)	**0.83 (0.74–0.91)**
Brain & eye	**0.65 (0.60–0.69)**	**0.68 (0.60–0.77)**
Thyroid	**0.88 (0.81–0.97)**	**0.78 (0.68–0.89)**
Bone	**0.88 (0.82–0.94**)	**0.65 (0.57–0.74)**
Hematopoietic	**0.70 (0.65–0.75)**	**0.53 (0.46–0.61)**
Others	0.92 (0.83–1.01)	**0.76 (0.67–0.87)**

Bold number units indicate a significant decrease in SBRs

Multivariate Poisson modelling, adjusting for calendar period, socioeconomic status, and year of diagnosis showed that parity status at diagnosis, cancer site, age at diagnosis, time since diagnosis and attained age were all independently and significantly associated with the probability of giving birth after cancer diagnosis (Table 4). Being parous at diagnosis, a diagnosis of prostate cancer, older attained age (41–70 years), diagnosis in childhood and adolescence (0–12 and 13–18 years) and a recent diagnosis (1–5 and 6–10 years) were significant and independent predictors of a reduced probability of giving birth after diagnosis. Men diagnosed in adulthood, increased time since diagnosis, cancer survivors aged 26–35 and survivors of thoracic and head and neck cancers were most likely to father an offspring.

**Table 4:** Poisson Regression model of relative likelihood of partners giving birth >1 year after cancer diagnosis of the men, expressed as Birth Rate Ratios (BRRs)

	Number of births	Person years	Unadj BRR (95 % CI)	BRR^a^ (95 % CI)
*Parity status at diagnosis*				
Nulliparous	5706	180421	1.00	1.00
Parous	2204	307707	**0.41 (0.39–0.43)**	**0.74 (0.69–0.79)**
*Cancer site*				
Melanoma skin	1032	55946	1.00	1.00
Head & neck	280	23671	**1.27 (1.11–1.45)**	**1.32 (1.15–1.50)**
Digestive	544	51622	**0.73 (0.66–0.81)**	0.99 (0.89–1.10)
Thoracic	91	9954	**2.28 (1.84–2.83)**	**1.83 (1.48–2.27)**
Breast	3	647	**3.22 (1.04–10.00)**	2.89 (0.93–8.98)
Prostate	4	30934	**0.04 (0.02–0.12)**	**0.25 (0.09–0.67)**
Reproductive organs	1555	60648	0.96 (0.89–1.04)	**0.69 (0.64–0.75)**
Brain & eye	1188	54827	**0.89 (0.82–0.97)**	**0.71 (0.65–0.77)**
Thyroid	674	35597	1.03 (0.94–1.14)	**0.90 (0.81–0.99)**
Bone	993	49411	1.05 (0.96–1.15)	**0.87 (0.80–0.96)**
Hematopoietic	935	47662	**0.89 (0.82–0.97)**	**0.69 (0.63–0.76)**
Others	611	67210	**0.60 (0.55–0.67)**	0.92 (0.83–1.01)
*Age at diagnosis*				
Adulthood (19 to 70 years)	891	24647	1.00	1.00
Adolescence (13 to 18 years)	6409	441720	1.24 (1.16–1.33)	**0.76 (0.69–0.83)**
Childhood (0 to 12 years)	610	21762	0.90 (0.83–0.98)	**0.44 (0.38–0.50)**
*Time since diagnosis*				
1 to 5 years	3738	226941	1.00	1.00
6 to 10 years	2090	111500	**1.31 (1.24–1.38)**	**1.10 (1.04–1.17)**
11 to 20 years	1558	105367	**1.23 (1.16–1.30)**	**1.23 (1.12–1.34)**
21 to 35 years	524	44322	**1.63 (1.49–1.79)**	**2.10 (1.77–2.50)**
*Attained age*				
16 to 25 years	682	31079	1.00	1.00
26 to 30 years	2160	26385	**3.52 (3.23–3.84)**	**2.74 (2.48–3.01)**
31 to 35 years	2448	35261	**3.15 (2.89–3.43)**	**2.38 (2.15–2.64)**
36 to 40 years	1572	43824	**1.88 (1.71–2.05)**	**1.44 (1.28–1.61)**
41 to 45 years	678	53092	0.90 (0.81–1.00)	**0.69 (0.61–0.80)**
46 to 70 years	370	298488	**0.31 (0.27–0.35)**	**0.25 (0.21–0.29)**

^a^All estimates are adjusted for parity status at diagnosis, cancer site, age and period of diagnosis, time since diagnosis, calendar period, attained age and socio-economic status. Bold number units indicate a significant difference in BRRs

Between 1961 and 2003, 126,696 children of the total 3,746,823 children were born to men who have had a diagnosis of cancer at any point in their lives (Fig. 1). These children were divided into groups based on the timing of their birth in relation to the diagnosis of their father’s cancer, where 115,938 children were born more than a year before their father’s cancer diagnosis, 2707 children born around the time of their father’s diagnosis and a further 8051 children born more than a year after their father’s diagnosis (Table 5). There was a total of 2604 deaths in these offspring, where 2500 (2.16 %) of the deaths occurred in children who were born more than a year before their father’s cancer diagnosis, 31 (1.15 %) deaths occurred in children born around the time of their father’s diagnosis and 73 (0.91 %) deaths occurred in children born more than a year after their father’s diagnosis.

**Table 5:** Timing of childbirth in relation to father’s cancer diagnosis for men born after 1931 and diagnosed with cancer between 1961–2003

	Child born *before* diagnosis^a^			Child born *around* diagnosis^b^			Child born *after* diagnosis^c^		
	Offspring	Deaths	(%)	Offspring	Deaths	(%)	Offspring	Deaths	(%)
Overall	115938	2500	2.16 %	2707	31	1.15 %	8051	73	0.91 %
Attained Age									
0–4 yrs old	–	1193	–	–	17	–	–	55	–
5–14 yrs old	–	238	–	–	4	–	–	5	–
15–24 yrs old	–	493	–	–	5	–	–	7	–
25+ yrs old	–	576	–	–	5	–	–	6	–
*Father's age at childbirth*									
16–25 yrs old	25153	696	2.77 %	324	4	1.23 %	668	6	0.90 %
26–30 yrs old	44543	1001	2.25 %	858	14	1.63 %	2175	17	0.78 %
31–35 yrs old	29547	541	1.83 %	732	4	0.55 %	2498	29	1.16 %
36–40 yrs old	11440	195	1.70 %	456	7	1.54 %	1614	12	0.74 %
41–45 yrs old	3690	55	1.49 %	186	2	1.08 %	707	6	0.85 %
46–70 yrs old	1565	12	0.77 %	151	0	0.00 %	389	3	0.77 %
*Mother's age at childbirth*									
12–20 yrs old	11600	342	2.95 %	142	1	0.70 %	216	2	0.93 %
21–25 yrs old	38170	947	2.48 %	613	8	1.31 %	1472	13	0.88 %
26–30 yrs old	40388	777	1.92 %	991	16	1.61 %	2810	22	0.78 %
31–35 yrs old	19301	333	1.73 %	666	3	0.45 %	2412	28	1.16 %
36–40 yrs old	5707	89	1.56 %	249	1	0.40 %	979	7	0.72 %
41–57 yrs old	749	10	1.34 %	44	2	4.55 %	155	1	0.65 %
Unknown	23	2	8.70 %	2	0	0.00 %	7	0	0.00 %
*Father's cancer site*									
Head & neck	5240	130	2.48 %	102	0	0.00 %	284	1	0.35 %
Digestive tract	21949	524	2.39 %	273	3	1.10 %	551	7	1.27 %
Thoracic	9847	268	2.72 %	75	0	0.00 %	94	1	1.06 %
Breast	184	4	2.17 %	2	0	0.00 %	3	0	0.00 %
Prostate	17998	433	2.41 %	14	0	0.00 %	4	0	0.00 %
Reproductive	4620	64	1.39 %	514	4	0.78 %	1599	12	0.75 %
Skin	9115	143	1.57 %	354	5	1.41 %	1045	12	1.15 %
Brain & eye	8047	137	1.70 %	353	1	0.28 %	1199	8	0.67 %
Thyroid	4231	104	2.46 %	159	1	0.63 %	687	8	1.16 %
Bone	11445	210	1.83 %	307	7	2.28 %	1010	7	0.69 %
Hematopoietic	7994	148	1.85 %	359	9	2.51 %	953	10	1.05 %
Others	15268	335	2.19 %	195	1	0.51 %	622	7	1.13 %
*Father's parity status at start of observation period*									
Nulliparous	113903	2444	2.15 %	996	13	1.31 %	5412	39	0.72 %
Parous	2035	56	2.75 %	1711	18	1.05 %	2639	34	1.29 %

^a^Children born >1 year prior to father’s cancer diagnosis

^b^Children born between 1 year before and 1 year after father’s cancer diagnosis

^c^Children born >1 year after father’s cancer diagnosis

Overall, mortality rate of offspring of fathers who have had a cancer diagnosis was similar to that of the background population (SMR 1.00, 95 % CI 0.96–1.04). This remained similar across all three groups of children, regardless of the timing of their birth in relation to their father’s cancer diagnosis (Table 6). There was no significant increase in mortality rate as the child grew older in all three groups of children. Variation in the father’s cancer site, age at diagnosis and age at childbirth did not cause a significantly higher mortality rate in the offspring compared to the background population. However, offspring of fathers in Group 3 who were parous at the start of observation period were shown to have a higher mortality rate compared to the background population (SMR 1.54, 95 % CI 1.06–2.10).

**Table 6:** Standardized Mortality Ratios (SMRs) of offspring born by partners of men with a history of cancer by timing of childbirth in relation to father’s cancer diagnosis

	Child born *before* diagnosis^a^	Child born *around* diagnosis^b^	Child born *after* diagnosis^c^
*Overall*	0.99 (0.96–1.03)	1.11 (0.75–1.53)	1.15 (0.90–1.43)
*Attained Age*			
0–4 yrs old	**0.91 (0.86–0.97)**	0.98 (0.57–1.50)	1.30 (0.97–1.66)
5–14 yrs old	1.10 (0.96–1.24)	1.41 (0.37–3.12)	0.77 (0.24–1.59)
15–24 yrs old	1.06 (0.97–1.16)	1.11 (0.35–2.29)	0.66 (0.24–1.29)
25+ yrs old	1.09 (1.00–1.18)	1.57 (0.50–3.25)	1.30 (0.47–2.55)
*Father's age at childbirth*			
16–25 yrs old	1.03 (0.96–1.11)	0.85 (0.22–1.90)	0.75 (0.27–1.47)
26–30 yrs old	**0.92 (0.86–0.97)**	1.28 (0.70–2.03)	0.85 (0.49–1.30)
31–35 yrs old	0.99 (0.91–1.07)	0.58 (0.15–1.30)	**1.53 (1.02–2.15)**
36–40 yrs old	**1.28 (1.10–1.46)**	2.05 (0.81–3.84)	1.18 (0.61–1.94)
41–45 yrs old	**1.48 (1.11–1.90)**	1.58 (0.15–4.54)	1.35 (0.43–2.80)
46–70 yrs old	1.02 (0.53–1.69)	–	1.80 (0.34–4.40)
*Father's cancer site*			
Head & neck	1.14 (0.95–1.34)	–	0.39 (0.00–1.54)
Digestive tract	1.07 (0.98–1.17)	1.09 (0.20–2.66)	1.55 (0.61–2.91)
Thoracic	**1.16 (1.03–1.30)**	–	1.23 (0.00–4.80)
Breast	1.09 (0.28–2.41)	–	–
Prostate	0.97 (0.88–1.06)	–	–
Reproductive	0.87 (0.67–1.10)	0.84 (0.22–1.87)	1.15 (0.59–1.90)
Skin	**0.82 (0.69–0.96)**	1.53 (0.48–3.16)	1.58 (0.81–2.60)
Brain & eye	0.89 (0.75–1.05)	0.25 (0.00–0.98)	0.75 (0.30–1.41)
Thyroid	**1.26 (1.03–1.52)**	0.63 (0.00–2.47)	1.42 (0.61–2.58)
Bone	0.88 (0.76–1.00)	2.16 (0.85–4.05)	0.82 (0.33–1.55)
Hematopoietic	0.90 (0.76–1.05)	2.01 (0.91–3.54)	1.20 (0.54–2.10)
Others	0.98 (0.88–1.09)	0.51 (0.00–2.00)	1.40 (0.56–2.64)
*Father's parity status at start of observation period*			
Nulliparous	0.99 (0.95–1.02)	1.17 (0.62–1.90)	0.95 (0.68–1.28)
Parous	**1.58 (1.20–2.03)**	1.07 (0.63–1.61)	**1.54 (1.06–2.10)**
*Father's age at diagnosis*			
0–12	–	–	1.25 (0.33–2.78)
13–18	–	–	0.95 (0.38–1.78)
19+	0.99 (0.96–1.03)	1.11 (0.76–1.54)	1.17 (0.89–1.48)

^a^Children born >1 year prior to father’s cancer diagnosis

^b^Children born between 1 year before and 1 year after father’s cancer diagnosis

^c^Children born >1 year after father’s cancer diagnosis

Bold number units indicate a significant difference in SMRs

After multivariate analysis with Poisson regression, adjusting for father’s age at childbirth, mother’s age at childbirth, father’s socioeconomic factor, attained age of the child and calendar period, the mortality risk of the offspring across all three groups of children were similar to the background population, suggesting that timing of birth of offspring in relation to the father’s diagnosis of cancer does not affect the child’s mortality risk (Table 7). However, increasing father’s age at childbirth and increasing mother’s age at childbirth were shown to be independent risk factors for increasing mortality risk of the offspring. Of note, children whose mothers were unknown were shown to be at an increased mortality risk. In this study, there was no significant difference between the causes of death (congenital anomalies, *p* = 0.172; perinatal conditions, *p* = 0.172; neoplasms, *p* = 0.368) and the timing of the birth of the offspring of fathers with or without a history of cancer (data not shown).

**Table 7:** Risk of death expressed as Mortality Rate Ratios (MRRs) in children born to partners of men with or without a history of cancer using a Poisson model

*Group*	Unadj MRR (95 % CI)	MRR^a^ (95 % CI)
Background Healthy Population	1.00	1.00
Group 1	**1.16 (1.11–1.20)**	1.01 (0.97–1.05)
Group 2	1.05 (0.74–1.50)	1.06 (0.75–1.51)
Group 3	1.03 (0.82–1.30)	1.10 (0.87–1.38)
*Father's age at childbirth*		
16–25 yrs old	1.00	1.00
26–30 yrs old	**0.88 (0.87–0.90)**	0.99 (0.96–1.01)
31–35 yrs old	**0.87 (0.85–0.89)**	**1.05 (1.02–1.09)**
36–40 yrs old	**0.90 (0.87–0.93)**	**1.15 (1.10–1.21)**
41–45 yrs old	0.95 (0.89–1.01)	**1.20 (1.11–1.29)**
46–70 yrs old	1.09 (0.98–1.22)	**1.33 (1.18–1.50)**
Mother's age at childbirth		
12–20 yrs old	1.00	1.00
21–25 yrs old	**0.85 (0.82–0.87)**	**0.94 (0.91–0.97)**
26–30 yrs old	**0.77 (0.75–0.79)**	**0.92 (0.89–0.95)**
31–35 yrs old	**0.80 (0.77–0.82)**	0.97 (0.93–1.02)
36–40 yrs old	**0.89 (0.85–0.94)**	**1.08 (1.02–1.15)**
41–57 yrs old	1.14 (1.01–1.28)	**1.34 (1.18–1.52)**
unknown	**5.51 (3.55–8.54)**	**4.96 (3.20–7.69)**

^a^Adjusted for father’s age at childbirth, mother’s age at childbirth, father’s socioeconomic factor, attained age of the child and calendar period. Bold number units indicate a significant difference in MRRs

## Discussion

This population based study shows that male cancer survivors are 23 % less likely to father an offspring compared to the background population. However, there is a large variation in birth rates depending on cancer site, age at diagnosis, parity status at diagnosis and time since diagnosis. Independent predictors for low birth rate following a cancer diagnosis were men who had prostate cancer, parous at diagnosis, recent diagnosis, diagnosed in childhood and aged over 40 years.

There are several reasons for the decreased birth rates among cancer survivors, contributed by the cancer itself, the side effects of systemic or locoregional treatment and psychosocial factors. There is a direct pathogenetic relationship between gonadal function and some malignancies such as testicular cancer. The adverse effects on gonadal function may also be mediated centrally through the effects on the hypothalamo-pituitary hormone axis. Chemotherapeutic agents, particularly alkylating agents (cyclophosphamide), procarbazineand platinum-agent (cisplatin) have been identified to be gonadotoxic [15, 16]. Radiotherapy with total-body irradiation and abdomino-pelvic irradiation can also affect reproductive function. The extent and reversibility of these gonadal effects tend to be dose dependent and vary by the age of the patient, type of cytotoxic regime [4, 8, 15] and the field, total dose and fractionation schedule of radiotherapy [17–20]. However, there remains a risk of permanent sterility.

A population based study conducted by Syse et al. in Norway had a similar finding to our study, where male survivors of cancer had a 24 % lower first birth rate compared to the background population of men without cancer of similar age, education in a similar period of time [21]. A separate study by Green et al. evaluated the long-term fertility of 6224 male survivors in the Childhood Cancer Survivor Study through questionnaires, which showed that male survivors were 44 % less likely to father a pregnancy than their siblings who had not undergone cancer treatment[8].

The diagnosis of cancer can also affect the survivors socially and psychologically to impact their attitude towards parenthood. With increased concerns of potential disease recurrence, concerns about dying and leaving their spouse to face single parenthood and not living long enough to see their children grow up and fear for the health of their offspring can impact negatively on their desire to have children [6, 22]. Also, cancer survivors are less likely to have a stable income and stable relationship with poorer job prospects and poorer health related quality of life; which can all affect their birth rates [23–26].

We observed very different fertility patterns for men with or without a child at diagnosis. Men who were parous at diagnosis had a significantly lower birth rate than those who were nulliparous, which is consistent with previously reported studies of fertility in cancer survivors [9, 21, 27]. This was reflected in all but one of the cancer sites (Breast Cancer) in our study. This finding is comparable to that in nulliparous female survivors of cancer who had similar birth rates to the background population in 8 out of 12 cancer sites [28]. This suggests that the true average ‘infertility’ in men following many types of cancers is only marginally less, if any, than the birth rates observed in cancer-free men. This finding is promising, as Mancini et al. showed that 33.5 % of fertile cancer survivors aged 20–44 desired to be parents and had the intention for additional children [29].

We identified several independent predictors of birth following a cancer diagnosis. These included parity status at diagnosis, cancer site, age at and time since diagnosis. Differences in birth rates based on age at diagnosis are likely related to the improvements in and increasing use of fertility preservation techniques, such as sperm banking and in-vitro fertilisation (IVF) in adolescents and adults diagnosed with cancer; as well as the increased toxicity of systemic treatment in children (0–12 years) and the inability to utilise sperm-banking for pre-pubertal males. Also men with prostate cancer are likely to be older and thus may have completed their family and no longer desire to have any children, which can contribute to a lower birth rate. The independent effect on increased birth rates of time since diagnosis may represent a physical and psychological perception of ‘cure’ from cancer, and return to normal function, where survivors may then be more able and willing to father a(nother) child.

Our study has also shown that offspring born to fathers who have cancer have similar mortality rates to the background population, and are not at an increased risk of death. This is consistent with previous studies which suggest that offspring of fathers who have had cancer have few other complications or adverse outcomes compared to the background population [30, 31]. This remained constant and was not affected by the timing of birth in relation to the cancer diagnosis. This is starkly different from mothers with cancer, where the timing of birth plays a major role, with offspring born around the time of diagnosis having a 66 % increased risk of death [32]. This is thought to be related to the intrauterine exposure to cancer treatment (chemotherapeutic agents and radiotherapy).

However, it is noted that offspring of parous men who were born after diagnosis had a higher mortality rate compared to the background population. This, coupled with the lower birth rates in men who were parous at diagnosis suggests that this particular group of men may need increased support, should they desire to have further offspring after their cancer diagnosis and treatment.

The mortality rate of offspring born around or after the time of diagnosis was not influenced by the attained age of the child, father’s socioeconomic status or the site of the father’s cancer. However, increasing father’s age at childbirth and increasing mother’s age at childbirth were shown to be independent risk factors for increasing mortality risk of the offspring. This is similar to the background population, where previous studies in a normal (non-cancer) population, have demonstrated a higher mortality rate in offspring of fathers aged 45 years or more [33] and mothers aged 35 years or more [34–36].

With over 80,000 men who were diagnosed with cancer who had over 120,000 offspring in a span of 40 years, our study is to our best knowledge, one of the largest studies evaluating the birth rates of male cancer survivors and the mortality rates of their offspring. The large population-based design allowed for an unbiased ascertainment of cancers, births and deaths. By calculating standardized birth ratios and standardised mortality ratios, we take into account societal changes in reproductive behaviour and mortality over time.

Weaknesses of our study include the lack of information of treatment details, which would have allowed for a better understanding of the observed trends. There was also absence of information on spontaneous or induced abortions in the partner/spouse, as well as the use of fertility preservation techniques which may have contributed to these findings. Furthermore, the measurement of fertility was implied through the birth rates of the subject’s partner/spouse, without considering possible paternal discrepancy or misattributed paternity. Any bias arising from this is difficult to predict as non-paternity rates vary greatly across populations (from 0.8 to 30 %) and have been associated with different demographic factors [37]. Our study included a substantial proportion of men who were treated decades ago and exposed to treatment regimens that may no longer be the standard of care. However, we observed no period effects, suggesting that there were no major changes in birth rates of the cancer survivors and mortality risks among their offspring over time. The study data was not extended beyond 2003 as more recent birth data among cancer survivors was not available.

## Conclusions

In summary, a diagnosis of cancer has an important impact on a man’s likelihood of fathering a child after the diagnosis. In this study, we observed the trends of changing birth rates in male Swedish cancer survivors, which are influenced by the site of cancer, age at and time since diagnosis. There is also a significant difference between survivors who are nulliparous vs. those who are parous at diagnosis, suggestive of a psychological element which may over-ride the physical ability of the survivor to have a(nother) child. However, offspring of male cancer survivors have a similar mortality rate to the background population, which is not influenced by the timing of their birth in relation to the cancer diagnosis, attained age of the child, father’s socioeconomic status or the site of the father’s cancer. Identifying men at increased risk of sub-fertility or infertility post-diagnosis, particularly those with a strong desire for parenthood; will allow for appropriate counselling and utilisation of fertility preservation techniques to give the men the possibility of parenthood after a cancer diagnosis.

## Abbreviations

BRR: birth rate ratios
CI: confidence interval
ICD7: International classification of diseases, Revision 7 code
ICSI: intracytoplasmic sperm injection
IVF: in-vitro fertilisation
MGR: multi-generation register
MRR: mortality rate ratios
SBR: standardized birth ratios
SMR: standardized mortality ratios
